# 

**Published:** 1858-02

**Authors:** 


### CONTENTS.

ORIGINAL COMMUNICATIONS— PAGE.

A Paper upon "Diseases of the Teeth." By W. W. Allport, D. D. S.... 53
Ulceration of the Os Uteri. By J. N. Graham, M. D........ .............. 70
Cases in Practice. By Charles Brackett, M. D.................... ............ 76
Advantages of the Prone Position in Examining the Fœtal Circulation as a Diagnostic Sign of Pregnancy. By W. H. Byford, M. D.......... 77

PROCEEDINGS OF MEDICAL SOCIETIES—

De Witt County Medical Society.............................................. 79
Organization of Medical Society of Waupaca County, Wisconsin.......... 83

BOOK NOTICES—

Prize Essay: Rational Therapeutics, or the Comparative Value of Different Curative Means, and the Principles of their Application. By Worthington Hooker, M. D............................................... 84

EDITORIAL—

Vexed Questions in Medical Ethics........................................ 88
Items from our Correspondents............................................. 94
Empyema Treated by Injections. By David Prince, M. D................ 95
American Medical Association.............................................. 96

ADVERTISEMENTS.......................................................... 97, 98, 99, 100

